# Anterior Knee Pain after Tibial Intra-medullary Nailing: Is it Predictable?

**DOI:** 10.5704/MOJ.1607.004

**Published:** 2016-07

**Authors:** PC Soraganvi, BS Anand-Kumar, R Rajagopalakrishnan, BA Praveen-Kumar

**Affiliations:** PES Institute of Medical Sciences and Research, Kuppam, Andrapradesh, India

**Keywords:** Tibia nailing, knee pain, tibial articular cartilage, patellartendon, intra medullary nail

## Abstract

**Introduction:** Intramedullary nailing has been used frequently for the treatment of tibial diaphyseal fractures. Chronic anterior knee pain has been considered the most frequent post-operative complication of this technique. We investigated the relationship between anterior knee pain and position of nail tip in proximal tibia.

**Methods:** 103 patients were selected among patients who underwent interlocking nailing in our institution. Patients with other factors that might cause anterior knee pain were excluded. In all patients intramedullary nailing was done using transpatellar approach. The patients were evaluated in two groups, 42 patients had anterior knee pain (Grup A), whereas 61 patients did not have pain (Group B). The distance from nail tip from tibial plateau was measured on lateral radiographs. Nail prominence from anterior tibial cortex was also measured.

**Results:** The two groups were similar with respect to gender and follow up period. Out of 42 patients who had knee pain 21 (50%) had nail tip within proximal third distance from plateau to tibial tuberosity. Twenty-four patients (42%) among knee pain group had nail prominence of more than 5mm from anterior tibial cortex followed by 12 patients (29%) within 5mm and 12 patients (29%) nail tip buried within the anterior cortex.

**Conclusion:** A greater incidence of knee pain was found when nail was prominent more than 5mm and when it is in the proximal third distance from tibial plateau to tuberosity. Patients should be aware of high incidence of knee pain when the nail tip is placed in proximal third and prominence of more than 5mm.

## Introduction

Intramedullary nailing is the most common treatment used for tibial shaft fracture management^1-5^. Many complications like non-union, infection, malunion, deep venous thrombosis, thermal necrosis and compartment syndrome are reported following tibial nailing, but are relatively low when compared with other procedures^4-6^. Chronic anterior knee pain is the most common problem associated with tibia nailing. In literature, incidence of knee pain after tibia nailing has been reported as high as 86%^7-9^. Multiple factors like skin incision, damage to intra-articular structures, gender, size of tibial platue, and presence of implant in medullary cavity have been reported to be the cause of anterior knee pain^6^. Often combinations of these factors are responsible for pain. Hence, it is difficult to predict based on single factor which patient is going to develop knee pain after tibia nailing.

Many studies have suggested prominence of nail as the major factor in the causation of anterior knee pain^4,10-12^. We find various suggestions regarding the correlation between position of nail and knee pain. In this study, we investigated the relationship of knee pain with the position of the nail tip in proximal tibia as seen in lateral radiograph.

## Materials and Methods

We retrospectively evaluated patients who underwent tibia intramedullary nailing for displaced tibia shaft fracture in our hospital from 2008 to 2012. Patients aged above 50 years, patients having other factors that might cause anterior knee pain like infection post operatively, broken implants, non-union, associated meniscal injury were excluded from the study. The information regarding presence of anterior knee pain was obtained retrospectively from clinical notes. A total of 103 patients were included in the study. These patients were divided into two groups, Group A with 42 patients having anterior knee pain and Group B consisting of 61 patients without knee pain. A few patients in whom the nail was removed because of anterior knee pain after fracture union were also included in Group A. Mean follow up in group A was 30 months (range 16 - 48 months) and in group B was 24 months (range 14 - 42 months).

All patients were operated by two trauma surgeons in our hospital using the same nail design. In all patients transtendinous approach was used for entry point. In all patients the nail was locked proximally and distally. Patient’s case notes were reviewed for details of surgery and for demographic data. In Group A there were 13 female and 29 male patients with mean age of 38, and in Group B there were 16 female and 45 male patients with mean age of 36. Measurement in radiograph was done by two resident doctors. As the measurements were done on digital radiograph, correction of magnification was not required. The observers were blinded and final measurement was taken as avereage of three measurements.

**Fig. 1:**
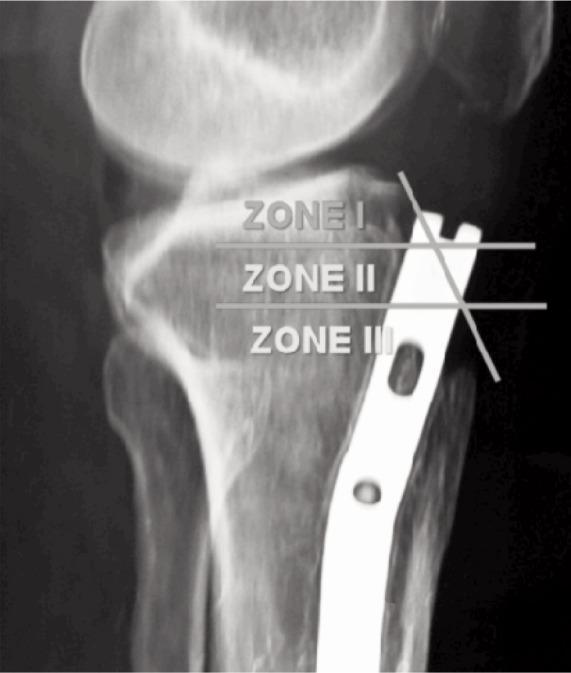
Lateral radiograph showing division of proximal tibia into three zones.

To evaluate the height of nail, the distance between the tibial articular surface and tip of tibial tuberosity in the lateral radiograph was divided into three equal zones by two horizontal lines (Fig 1). The height of the nail was defined in the lateral radiograph as the distance between a line drawn through tibial plateau and a parallel line to this line touching the apex of the nail^12^. These zones were named as zone I, zone II and zone III from top to bottom respectively. The height of the nail was noted in both groups by marking position of the nail tip with respect to these zones on lateral radiograph.

**Fig. 2:**
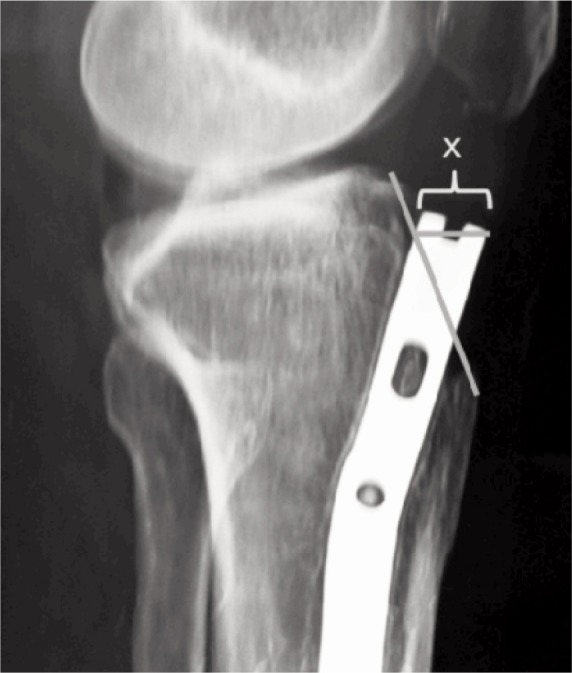
Measurement of anterior cortex nail distance.

The anterior cortex nail (ACN) is the distance between the anterior cortex of tibia and the anterior nail tip^12^. It was measured by drawing a line over the anterior cortex of tibia and measuring the distance in millimeters between this line and anterior tip of nail (Fig 2). Based on the measurements, the patients were divided into three groups. The first group with ACN more than 5mm, second group with ACN between 0mm to 5mm and the third group where ACN had a negative value, which meant that the nail tip was burried within the anterior cortex in the lateral radiograph.

Statistical analysis was performed using SPSS version 19. Results were represented in percentage and proportion. The association between nail position and anterior knee pain was assessed using Pearson Chi-square Test and the probability (P) value less than 0.05 was considered statistically significant.

## Results

One hundred and three patients were included in the study, age ranging between 18 years to 49 years. Out of the 42 patients in Group A, visual analogue score (VAS) was noted between 4 to 7. In this group the majority of patients complained of knee pain while kneeling. Thirty patients (71%) had pain while kneeling which is followed by other activities like walking, climbing stairs, squatting, and sitting crossed leg. Four patients had pain at rest (10%).

### Height of nail (Table I)

**Table I:** Distribution of patients in three zones

	Group A(pain)	Group B (no pain)	
Zone I	21	16	37
	(50%)	(26%)	
Zone II	11	15	26
	(26%)	(25%)	
Zone III	10	30	40
	(24%)	(49%)	
Total	42	61	103

In Group A, 21 out of 42 patients (50%) had nail tip in zone I followed by 11 (26%) in zone II and 10 (24%) in zone III. In Group B, 39 patients (49%) had nail tip in zone III followed by 16 patients (26%) in zone I and 15 patients (25%) in zone II.

The difference was found to be statistically significant (Pearson Chi-square 8.06, Degrees of freedom 2, P=0.018).

### Anterior cortex-nail distance (Table II)

**Table II:** Distribution of patients with respect to ACN distance

	**Group A(pain)**	**Group B (no pain)**	
< 0mm	8	14	22
	(19%)	(23%)	
0mm t0 5mm	10	41	51
	(24%)	(67%)	
> 5 mm	24	6	30
	(57%)	(10%)	
	42	61	103
	(100%)	(100%)	

**Table III:** Distribution of patients in Group B with respect to zones and ACN distance

	**< 0 mm**	**0 mm to 5 mm**	**> 5mm**	
Zone I	5	2	14	21
Zone II	2	3	6	11
Zone III	1	5	4	10
	8	10	24	42

Twenty-four patients (57%) in Group A had ACN distance of more than 5mm, followed by ten patients (24%) within 5mm and eight patients (19%) with nail tip buried within the anterior cortex. Fourty-one patients (67%) in Group B had ACN distance within 5mm, 14 patients (23%) buried within cortex. Only six patients (10%) had ACN of more than 5mm.

The difference was found to be statistically significant (Pearson chi square 28.753, Degree of Freedom 2, P=0.000).

## Discussion

Anterior knee pain is a common complication of intramedullary nailing for tibia fractures^4^. Many factors like damage to articular surface and meniscus, injury to the infrapatellar branch of the saphenous nerve, infrapatellar fat pad, nail prominence, thigh muscle weakness, and small plateau width contribute to the pain^4,11^. Incidence of anterior knee pain has been noted between 31% to 86% in various studies^13-20^. Fourty-two patients (41%) developed anterior knee pain in this study which is comparable to other similar studies.

Present litrature is not clear about correlation between type of surgical approach and knee pain. No study has provided definitive evidence of decrease in knee pain using a single approach. From available literature and meta-analysis, it is evident that paratendinous and transtendinous approaches for entry point do not make any differences cause of anterior knee pain^11,9,21^. All patients included in this study were operated by the transtendinous approach.

The protrusion of nail tip (anterior and superior prominence) has been reported as one of the contributing factors for knee pain^10^. Keating *et al* in their study had observed that anterior knee pain was more related to ACD (of more than 5mm) rather than the height of nail^10^. However, Bhattacharyya *et al* reported that both anterior and superior nail prominence caused pain^11^. In their study the greater incidence of rest pain was seen in anterior nail prominance, whereas superior nail prominence was associated with pain while kneeling^7,11^.

**Fig. 3:**
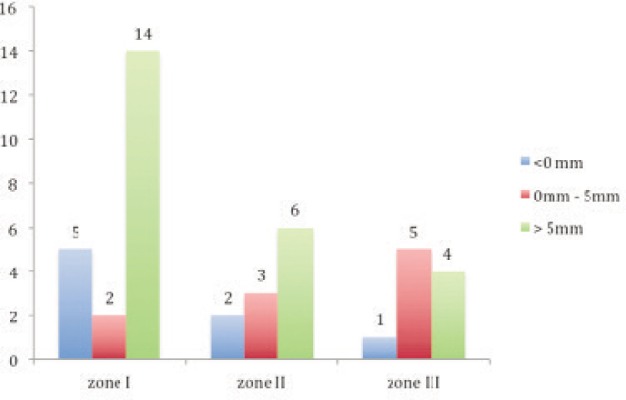
Distribution of patients in Group A (patients with pain) with respect to zone and ACN distance.

We observed that half of the patients with knee pain in our study had nail tip prominent superiorly (50% in zone I) and ACD was more than 5mm (57%) [Fig 3]. These correlations were statistically significant. Hence, we recommend burying of the nail tip to avoid knee pain. However, it cannot be concluded that ACD is correlated more with anterior knee pain than superior prominance as there was marginal difference in incidence.

Most of the patients in our study complained pain while kneeling, sitting crossed legged and squatting, irrespective of superior or anterior nail prominence. Pain may be due to irritation of patellar tendon by the protruded nail as these activities required high knee flexion. In a few patients knee pain was noted in spite of the nail tip being not prominent. The causes of knee pain are multifactorial and protruded nail alone cannot be blamed.

Many intra-articular and extra articular structures are believed to be responsible for knee pain after tibia nailing^22-24^. Hernigou *et al* in their study noticed that structures like medial meniscus, lateral tibial plateau and transverse ligament were at risk of damage while making entry point. Hence they recommend entry point anterior to the transverse ligament and anterior horn of each meniscus^23^. McConnell *et al,* based on their study on cadaveric knees recommended entry point medial to the lateral tibial spine and anterior to articular margin to avoid articular cartilage damage^24^. Both these studies suggest that the closer the entry point was to the tibial articular surface there was greater risk of damage to the intra-articular structures. This is evident in the current study (as more half of patients in Group A had nail tip in zone I). Hence, we recommend the burying of the nail tip up to zone II.

It is evident that the smaller size of the tibial plateau is associated with knee pain^11^. The tibial plateau size is also variable among different patients. Hence, while measuring the height of nail we prefered to divide it into zones rather than taking linear measurement. We observed that the height of nail correlated with anterior knee pain as half of the patients with knee pain had tip of nail in zone I, close to the tibial articular surface. This relationship was found statistically significant. We believe that injury to adjacent structures like synovium, meniscus, intermeniscus ligament or articular cartilage damage may contribute to the pain.

Our study suggests that anterior nail prominence of more than 5mm and superior prominence of nail closer to tibial articular surface are associated with anterior knee pain. Hence we recommend extra-articular entry point, burying the nail tip and avoiding nail tip proximity to tibial plateau.
